# Gender Differences in Objective and Subjective Measures of ADHD Among Clinic-Referred Children

**DOI:** 10.3389/fnhum.2019.00441

**Published:** 2019-12-13

**Authors:** Ortal Slobodin, Michael Davidovitch

**Affiliations:** ^1^Department of Education, Ben-Gurion University, Beer-Sheva, Israel; ^2^Medical Department and Research Institute, Maccabi Healthcare Services, Tel Aviv, Israel

**Keywords:** attention, ADHD, CPT, distractibility, gender, impulsivity

## Abstract

Attention deficit hyperactivity disorder (ADHD), one of the most prevalent childhood disorders today, is generally more likely to be diagnosed and treated in boys than in girls. However, gender differences in ADHD are currently poorly understood, partly because previous research included only a limited proportion of girls and relied mainly on subjective measures of ADHD, which are highly vulnerable to reporter’s bias. To further examine gender differences in ADHD and to address some of the shortcomings of previous studies, this study examined gender differences in subjective and objective measures of ADHD among clinic-referred children with ADHD. Participants were 204 children aged 6–17 years-old with ADHD (129 boys, 75 girls). A retrospective analysis was conducted using records of a clinical database. Obtained data included parent and teacher forms of the Conners ADHD rating scales, Child Behavior Checklist (CBCL), Teacher’s Report Form (TRF), and child’s continuous performance test (CPT) scores. Results showed that according to parents’ and teachers’ reports of ADHD-related symptoms (Conners ADHD rating scales), girls had more inattention problems than boys, but no differences were identified in the level of hyperactivity and impulsivity symptoms. CPT data, however, revealed higher impulsivity among boys. We did not find gender differences in the level of distractibility during CPT performance. Specifically, the effects of distractors type (visual environmental stimuli, auditory stimuli, or a combination of them) and distractors load (one or two distracting stimuli at a time) on CPT performance did not differ between boys and girls with ADHD. These findings suggest that gender effects on ADHD symptoms may differ between subjective and objective measures. Understanding gender differences in ADHD may lead to improved identification of girls with the disorder, helping to reduce the gender gap in diagnosis and treatment.

## Introduction

Attention deficit hyperactivity disorder (ADHD) is one of the most prevalent childhood disorders today (Barkley, 2015), with an estimated worldwide prevalence of 7.2% in children under 18 years of age (Thomas et al., 2015). Although gender has been considered a significant factor in ADHD research for many years (Arnold, 1996), gender differences among children with ADHD are not well understood (Hasson and Fine, 2012). Generally, boys are more likely to be referred, diagnosed, and treated for ADHD symptoms than girls. These findings were previously attributed to gender differences in the manifestation of ADHD (e.g., males having more disruptive symptoms; Gaub and Carlson, 1997; Gershon, 2002) as well as to referral bias (Rucklidge, 2008, 2010; Ohan and Visser, 2009). Understanding the role of gender in ADHD care has been historically hindered by methodological issues, such as involving relatively low numbers of girls in research samples, failing to control for possible gender effects, and relying solely on subjective scales which are often subjected to reporter’s bias (Quinn and Madhoo, 2014). As a result, literature focusing on ADHD in female subjects, and gender differences in ADHD has been limited (Nadeau and Quinn, 2002; Sassi, 2010). The aim of the current study was, therefore, to examine gender differences in ADHD-related symptoms, using subjective and objective measures, within a clinic-referred sample of 6–17-year-old children with ADHD.

## Gender Differences in ADHD Prevalence

Research has consistently shown that boys are more likely to be diagnosed and treated for ADHD-related symptoms than girls (Biederman et al., 2002; Gudjonsson et al., 2014). Male-to-female ratios of ADHD diagnosis ranged from 2:1 to 10:1 (Nøvik et al., 2006; Ramtekkar et al., 2010; Willcutt, 2012), with higher male-to-female ratios found in clinical vs. population-based samples (Skogli et al., 2013).

Research on gender differences in ADHD suggests that girls may be consistently under-identified and underdiagnosed because of differences in the disorder manifestation among boys and girls. Girls diagnosed with ADHD show fewer hyperactive/impulsive symptoms and more inattentive symptoms when compared with boys with the disorder (Biederman et al., 2002; Biederman and Faraone, 2004). Further, girls with ADHD present more commonly with the inattentive subtype than do boys (Hinshaw et al., 2006). In addition, males with ADHD have been found to have more co-existing externalizing disorders (conduct disorder, oppositional defiant disorder) and symptoms (e.g., aggression, rule-breaking) than typically developing boys, while females tend to show more internalizing disorders (e.g., anxiety) in comparison to typically developing girls (Biederman et al., 2010; Hinshaw et al., 2012). Symptoms of inattention and internalization might be less likely to be disruptive in the classroom, resulting in fewer referrals, diagnoses, and treatment of ADHD in girls (Biederman et al., 2002; Diamantopoulou et al., 2007). In a recent Swedish large-scale study, Mowlem et al. (2019) showed that hyperactivity/impulsivity symptoms, as well as conduct problems, were stronger predictors of clinical diagnosis and prescription of pharmacological treatment than other types of ADHD-related symptoms, suggesting that females with ADHD may be more easily missed in the ADHD diagnostic process and less likely to be prescribed medication unless they have prominent externalizing problems.

Another possible reason for underdiagnosis and undertreatment of ADHD in girls is that symptoms of inattention are more likely to be present in a structured educational environment, such as in high school or college, which may delay diagnosis among females (Bruchmüller et al., 2012). Lastly, females with ADHD may develop better-coping strategies than males to compensate for their ADHD-related difficulties, such as working hard to maintain classroom performance. As a result, they can better mitigate or mask the impact of their difficulties (Quinn, 2010). In addition to the gender discrepancies in the expression of ADHD, referral bias may account for the gender differences in ADHD prevalence. Many studies demonstrated that parents, teachers, and professionals are more likely to recognize ADHD-related symptoms in boys than in girls and are more likely to refer boys to treatment (Glass and Wegar, 2000; Bruchmüller et al., 2012). For example, Papageorgiou et al. (2008) conducted a study that collected parent and teacher reports of ADHD behaviors of children and measured the agreement of the parent and teacher reports. The results showed that parents rated boys higher on the hyperactivity scale than girls, but not on emotional problems, conduct problems, and peer problems. Teachers rated boys higher on inattention, hyperactivity, and conduct problems than girls. Likewise, teachers were more likely to refer boys for ADHD treatment, even when showing equal or lower levels of impairment compared to girls (Sciutto et al., 2004; Coles et al., 2012). When gender differences were assessed in a sample of non-referred children (Biederman et al., 2005), boys and girls did not differ in subtypes of ADHD, psychiatric comorbidity, or treatment history. Girls also showed similar levels of cognitive, school, and family functioning. The authors concluded that the clinical correlates of ADHD are not influenced by gender and that gender differences observed in clinical settings may be caused by referral biases.

## Gender Differences in a Continuous Performance Test (CPT)

Usually, ADHD diagnosis in children and adolescents involves multiple sources of information, including clinical examination, parents’ and teachers’ reports and self-report scales (Wolraich et al., 2011). The vulnerability of these methods to clinicians and informant biases (Rousseau et al., 2008) may lead to underdiagnosis or overdiagnosis of ADHD, not only in girls but also in other groups such as ethnic minorities (Lambert et al., 2002). Given the limited validity of subjective measures of ADHD, there has long been an interest in using objective, laboratory-based tools that could provide a norm-referenced measure of ADHD. The CPT is the most frequently used direct measure of ADHD-related inattention, impulsivity, and hyperactivity (Vogt and Williams, 2011). Typically, the test includes a rapid presentation of a sequence of visual or auditory stimuli (numbers, letters, number/letter sequences, or geometric figures). Participants are instructed to respond to the “target” stimulus and to avoid responding to “non-target” stimuli. Responses to non-target stimuli are referred to as “commission errors,” and are considered as a measure of impulsivity. An absence of response to target stimuli is referred to as an “omission error” and is assumed to measure inattention. Other common measures of CPT responses include the number of correct responses, response time (RT), and the variability in RT.

The influence of gender on CPT performance among children with ADHD is not clear. Some studies had shown girls to have fewer CPT errors, superior signal detection, and less inattention with longer interstimulus intervals (Arnold, 1996). Other studies, however, failed to identify the gender difference in CPT performance (Yang et al., 2004). A meta-analysis of gender differences in CPT among clinic-referred children indicated that consistent with rating scale studies (Gaub and Carlson, 1997; Gershon, 2002), boys with ADHD committed significantly more commission errors than girls with ADHD. However, no gender differences were found in the rate of omission errors. These findings suggest that inhibitory control, but not attention deficit may be mediated by gender. Alternatively, the lack of gender differences in inattention may be attributed to methodological limitations of the included studies, mainly the inclusion of a low number of studies, which were based on predominantly male samples (Hasson and Fine, 2012).

## The Current Study

The gender gap in clinical populations of children with ADHD continues to hinder the correct diagnosis and treatment of girls with the disorder (Skogli et al., 2013). Thus, understanding how gender influences ADHD manifestations may have important clinical, ethical, and public implications. Prior studies have shown that girls with ADHD are under-identified due to sex-specific biases and expectations (Waschbusch and King, 2006; Meyer et al., 2017) and that the threshold for referral and diagnosis of ADHD in girls might be higher than for boys (Mowlem et al., 2019). While these studies were able to identify gender differences on standardized rating scales, differences in gender performances on direct CPT measures have received less attention (Hasson and Fine, 2012). The current study sought to assess gender differences in rating and objective measurements of ADHD as well as in co-occurring problems in a clinic-referred sample of children with ADHD. Based on a relatively balanced female-to-male ratio (1:1.7), the current study examined the gender differences in parent and teacher ADHD rating scales, co-occurring symptoms, and CPT performance indices (attention, timing, impulsivity, and hyperactivity). In addition, we examined gender differences in the level of distractibility and in time-on-task effects during CPT performance. Although increased distractibility is considered one of the core symptoms of ADHD within the inattention domain (American Psychiatric Association, 2013), direct and systematic research on this deficit and how it is differently patterned in males and females is currently very limited. Addressing gender differences in objective and subjective measures of ADHD, as well as in co-occurring symptoms may overcome some of the clinician’s and reporter’s gender-related biases observed in ADHD rating scales. Furthermore, using different types of measures would increase our understanding of ADHD underdiagnosis in females and whether certain symptoms are more predictive of ADHD referral and diagnosis in males than in females (or vice versa).

## Materials and Methods

### Participants and Procedure

Israel has a socialized healthcare system in which all citizens are free to choose between four health maintenance organizations (HMOs). Patient fees are equivalent across all four HMOs, and all HMOs provide equivalent medical services that are based on national health regulations. The diagnosis of ADHD in Israel is usually given by a psychiatrist or a neurologist and includes the use of the Diagnostic and Statistical Manual of Mental Disorders (DSM) criteria and a formal diagnostic questionnaire for parents and teachers (Hezi, 2010).

The current study included 204 children diagnosed with ADHD (63% boys), referred to an outpatient pediatric neurologic clinic, affiliated with the second-largest HMO. Children were referred to the clinic for ADHD evaluation between January 2014 and December 2017. Participating children and their families were all of Jewish background, lived in rural and urban areas in Northern Israel, and had medium-high or high socioeconomic status, based on a social scale that divides geographic locations into different socioeconomic categories (Israeli Central Bureau of Statistics, 2017).

Children’s age ranged between 6 and 17 years (Mean age = 9.44, SD = 2.42). No age differences were found between girls and boys (*t*_(203)_ = 1.01, N.S).

Inclusion criteria were children between 6–17 years, diagnosed with ADHD. The diagnostic procedure was conducted by a certified pediatric neurologist and included an interview with the child and parents, medical/neurological examination, CPT administration, and ADHD diagnostic questionnaires.

Diagnosis of ADHD was considered positive if, based on both parents’ and teacher’s reports (Conners, 2008), the child scored above the standard clinical cut-offs for ADHD symptoms. Since this is a clinical setting, a more conservative cut-off (+2 Standard deviations and above) for ADHD diagnosis was used (Barkley, 2015).

Exclusion criteria were an intellectual disability, chronic neurological levels (e.g., cerebral palsy, autism spectrum disorder), and psychosis. The protocol for the research project conforms to the provisions of the Declaration of Helsinki, approved by the Institutional Review of Board of Maccabi health services.

### Measurements

Background variables included the child’s age and gender, ethnicity, socio-economic status, place of residence, and school type.

ADHD-related symptoms were assessed by the parent and teacher forms of the Conners ADHD Index Rating scales, 3rd edition, short-form (Conners 3 AI; Conners, 2008), Hebrew version (Psychtech Ltd, 2012). The Conners 3 is a multi-informant assessment of children between 6 and 18 years of age that takes into account home, social, and school settings and is considered to be a reliable instrument for detecting ADHD problems in children aged 6–18 years.

Co-existing psychiatric symptoms were measured by the Child Behavior Checklist (CBCL), and the Teacher’s Report Form (TRF; Achenbach and Rescorla, 2001), Hebrew version (Psychtech Ltd, 2005). These forms include eight DSM-oriented scales consistent with DSM diagnostic categories: Anxious/Depressed, Withdrawn/Depressed, Somatic Complaints, Social Problems, Thought Problems, Attention Problems, Rule-Breaking Behavior, and Aggressive Behavior.

CPT performance—the study employed the MOXO-CPT^1^ version (Berger and Goldzweig, 2010), a standardized computerized test designed to diagnose ADHD-related symptoms. Like other CPTs, the MOXO-CPT measures sustained attention, omission and commission errors, and RT. However, as detailed below, it differs from other CPTs in its ability to differentiate between different types of disinhibited responses and between problems in RT and inattention. Importantly, the test incorporates external interfering stimuli (auditory and visual) serving as measurable distractors, a feature that is unique to the MOXO-CPT. The test’s validity and utility in distinguishing children and adolescents with ADHD from their typically developing peers were demonstrated in previous studies (Berger and Cassuto, 2014; Berger et al., 2017; Shahaf et al., 2018).

#### General Description

The test included eight levels (stages); each consisted of 53 trials (33 target and 20 non-target stimuli) and lasted 114.15 s. The total duration of the test was 15.2 min. On each trial, a stimulus (target or non-target) was presented in the middle of the screen for 0.5, 1, or 3 s and was followed by a “void” of the same length (Supplementary Figure S1). Each stimulus remained on the screen for the full presentation time, regardless of whether a response was provided or not. This practice allows the measuring of RT as well as its accuracy. The child was instructed to respond to the target stimulus as quickly as possible by pressing the space bar once and only once. In addition, the child was instructed not to respond to any other stimuli but the target, and not to press any other key but the space bar.

#### Test Stimuli

Target and non-target stimuli were cartoon pictures. Given that ADHD often co-occurs with specific learning disabilities that may be confounded with CPT performance, all stimuli were free of letters or numbers (Seidman et al., 2001). The target stimulus was always a cartoon image of a child’s face. Non-target stimuli included five different images of animals.

#### Distracting Stimuli

To improve the test’s ecological validity and to simulate the everyday environment, the MOXO-CPT incorporated visual and auditory distracting stimuli that were not part of the non-target stimuli. Distractors’ onset was not synchronized with the onset of the target or the non-target stimuli.

Distractors were short animated video clips with typical elements of the child’s everyday life. Overall, six different distractors were presented, each of them could appear as pure visual (e.g., birds moving their wings), pure auditory (e.g., birds singing), or as a combination of visual and auditory stimuli (birds singing and simultaneously moving their wings). Distractor presentation time varied between 3.5 and 14.8 s, with a fixed interval of 0.5 s between two distractors. There were six various visual distractors: a bowling ball (presented for 3.5 s), warrior (Jedi) with a saber, a gong (6.8 s), birds (9.25 s), (14.8 s), saber (6.8 s), and a flying airplane (8.6 s). Auditory distractors included the six corresponding sounds of the visual distractors.

#### Test Levels

The test included eight levels, each included different distractors set: Levels 1 and 8 did not include any distractors. Levels 2 and 3 included pure visual stimuli, levels 4 and 5 included pure auditory stimuli, and levels 6 and 7 included a combination of visual and auditory stimuli. During levels 2, 4, and 6, only one distractor was presented at a time. During levels 3, 5 and 7, two distractors were presented simultaneously.

Performance indices—The MOXO-CPT measured four performance indices:

(1) Attention: the number of correct responses (pressing the key in response to a target stimulus), which were conducted either during the stimulus presentation or during the void period that followed. This method allows the test to evaluate whether the participant responded correctly to the target (was attentive to the target) independently of his/her RT. The number of omission errors were also calculated (i.e., the number of times that the patient did not respond to a target stimulus). The score in the Attention index was calculated as the average of correct responses throughout the eight test levels.
(2) Timing: the number of correct responses (pressing the key in response to a target stimulus) that were given while the target stimulus was still presented on the screen. This index excluded responses that were performed during the void period (after the stimulus has disappeared). This method allowed the test to differentiate between the overall rate of correct responses (measured by the Attention index) and the rate of correct responses that were given only on the right timing (measured by the Timing index). These two aspects of RT correspond to two different deficits typical to ADHD: difficulty to provide an accurate response and difficulty to respond on time (National Institute of Mental Health, 2012). The score in this index was calculated as the average of correct responses while the target stimulus was still presented on the screen throughout the eight test levels.
(3) Impulsivity: the number of commission errors performed only when a non-target stimulus was present on the screen. Other types of non-inhibited responses (e.g., pressing the keyboard more than once) were not considered as impulsive responses (as will describe in the next paragraph). Score in this index was calculated as the average of impulsive responses throughout the eight test levels.
(4) Hyperactivity: the total number of commission responses that were not coded as impulsive responses (e.g., multiple responses, random key pressing). Differentiating between commission errors that were conducted due to impulsive behavior and commission errors that were conducted due to motor hyper-responsivity allowed the identification of multiple sources of response disinhibition. The score in this index was calculated as the average of hyperactive responses in the eight test levels.

The MOXO-CPT version for adolescents and adults, that was administered to participants aged 13 and above, differed from the children’s version in several aspects. First, in each trial, the stimulus (target/non-target) is presented for 0.5, 1 or 4 s, followed by a “void” period of the same duration. Second, eight different distractors were used instead of six. Distractors were based on adults’ and adolescents’ everyday life, including car driving, a crying baby, and arguing people. Third, all distractors were presented for 8 s, with a fixed interval of 0.5 s between two distractors. Finally, each level consisted of 59 trials (34 targets and 25 non-targets) and lasted 136.5 s, so that the total duration of the test was longer (18.2 min). All other test’s characteristics were identical to the children’s version.

### Data Analysis

To address gender differences in teachers’ and parents’ rating scale of child’s behavior (TRF and CBSL, respectively), we performed multivariate analysis of variance with covariates (MANCOVA). In these analyses, gender served as the independent variable, and the eight CBCL or TRF subscales were used as dependent variables.

To examine gender differences in teachers’ and parents’ reports of ADHD symptoms (according to the Conners rating scales for teachers and parents, respectively), we performed two-way repeated-measures ANOVA. Symptom type (inattention or hyperactivity/impulsivity) and informant role (teacher or parent) were the within-subject factors, and gender was the between-subject factor. Of interest were the interaction effects of gender * symptom type, gender * informant role, and the three-way interaction (gender * symptom type * informant role). These effects may provide evidence that gender differences in ADHD symptoms vary as a function of symptom type, informant role or both.

Gender differences in the agreement rates between teacher and parents rating of ADHD-related symptoms were examined with chi-square tests.

Further, we examined gender differences in the four MOXO-CPT performance indices, using a one-way repeated measures ANOVA, with test levels as the within-subject factor and gender as the between-subject factor. In addition, we examined gender effects on the difference between the first and the last test levels (for each CPT index) in order to explore whether boys and girls are differently affected by time on the task.

Finally, to examine gender differences in distractibility levels during CPT performance, we first calculated the difference between the mean score in the no-distractor level (base-line) and the mean score in each distractor type (pure visual, pure auditory, and a combination of visual and auditory distractors). This calculation was conducted separately for each CPT index. The outcome of this calculation is considered a measure of the distractibility level. Next, we conducted two-way repeated-measures ANOVA. Distractor type (visual, auditory, or combined) and distractibility load (low or high distractibility) was the within-subject factors, and gender was the between-subject factor. Of interest were the two-way interactions (gender * distractor type and gender * distractibility load) as well as the three-way interaction (gender * distractor type * distractibility load). Such interactions would provide evidence for differential patterns of sensitivity to environmental distractors between boys and girls. Participants’ age served as a covariate in all analyses. Power analysis calculation revealed that using a two-tailed test, *α* = 0.05, and power = 0.80 (Cohen, 1992), a minimum of 51 participants is required in each gender group. Thus, our sample size (*N* = 204) was able to provide adequate power to detect a medium effect size (Cohen *d* = 0.5). All multivariate analyses were followed by posthoc analyses with Bonferroni correction for multiple comparisons. Analyses were conducted with SPSS software for Windows Version 25 (SPSS, Inc., Chicago, IL, USA).

## Results

### Gender Differences in Co-existing Symptoms

To test gender differences in parents’ and teachers’ reports of child’s behavior we conducted a one-way MACNOVA for the CBCL and the TRF. The total scores in the CBCL and the TRF were the dependent variables, and gender was the independent variable. Age served as a covariate variable. The results of the analyses are presented in Table 1.

**Table 1:** Parent and teacher rating of child’s behavior, by gender.

		Boys (*n* = 129)		Girls (*n* = 75)		Gender differences
		Mean score	SD	Mean score	SD	
**CBCL parent report**
Anxious/depressed		59.35	8.11	60.49	8.511	*F*_(8,192)_ = 1.62, *p* = 0.12
Withdrawn/depressed		57.10	8.54	55.56	8.896
Somatic complaints		57.60	8.99	57.33	8.034
Social problems		58.81	7.42	60.13	9.002
Thought problems		63.52	63.61	57.70	7.649
Attention deficit		64.71	7.91	67.53	8.270
Rule-breaking behavior		57.81	7.60	56.87	6.705
Aggressive behavior		62.89	9.14	62.60	9.614
**TRF teacher report**					
Anxious/depressed		61.75	8.91	59.08	7.62	*F*_(8,192)_ = 6.32, *p* < 0.001
Withdrawn/depressed		58.95	7.24	58.25	8.14
Somatic complaints		56.98	7.68	55.96	7.15
Social problems		60.61	8.30	61.17	8.44
Thought problems		59.59	7.19	58.12	7.69
Attention deficit and hyperactivity		62.76	5.19	64.77	5.26
Rule-breaking behavior		59.74	7.69	56.05	6.65
Aggressive behavior		64.48	10.26	62.84	8.01
**Conners rating scales**					
Parent rating of inattention		72.11	11.13	76.25	9.01	*F*_(1,200)_ = 10.04, *p* = 0.002
Teacher rating of inattention		71.12	7.76	76.55	8.08
Parent rating of hyperactivity/impulsivity		73.47	14.00	76.43	14.26
Teacher rating of hyperactivity/impulsivity		71.26	12.72	70.88	13.81
Parent-teacher agreement on inattention symptoms	No report	5	3.8	0	0	*χ*^2^_(2, *N* = 204)_ = 12.08, *p* = 0.002
	Parent or teacher	38	29.4	9	12
	Both parent and teacher	86	66.6	66	88
Parent teacher agreement on hyperactivity/impulsivity symptoms	No report	15	11.6	8	10.7	*χ*^2^_(2, *N* = 204)_ = 2.26, *p* = 0.88
	Parent or teacher	37	28.7	24	32
	Both parent and teacher	77	59.6	43	57.3	

*Note. Higher scores mean greater pathology*.

Overall, MANCOVA results of the CBCL subscales indicated that the effect of gender was not significant, Wilks’ Lamda = 0.937, *F*_(8,192)_ = 1.62, *p* = 0.12. MANCOVA results of the TRF subscales yielded a significant overall main effect of gender, Wilks’ Lamda = 0.792, *F*_(8,192)_ = 6.32, *p* < 0.001. Univariate comparisons revealed a main effect for gender so that according to teachers’ reports, boys had more anxiety/depression symptoms, *F*_(1,199)_ = 4.81, *p* = 0.03, and more rule-breaking behaviors, *F*_(1,199)_ = 11.89, *p* = 0.001 than girls. Girls, on the other hand, were more likely to present attention difficulties/hyperactivity symptoms, *F*_(1,199)_ = 5.96, *p* = 0.02. Because inattention and hyperactivity are included in the same subscale of the TRF, it was impossible to identify whether teachers perceived girls as more inattentive or more hyperactive than boys.

### Gender Differences in Parent and Teacher ADHD Rating Scales

To examine gender differences in teachers’ and parents’ reports of ADHD symptoms (according to the Conners rating scales for teachers and parents, respectively), we performed two-way repeated-measures ANOVA. Symptom type (inattention or hyperactivity/impulsivity) and informant role (teacher or parent) were the within-subject factors, and gender was the between-subject factor. Table 1 summarizes gender differences in Conners’s ADHD scores according to parents’ and teachers’ reports. Analyses did not find a main effect for symptom type, Wilks’ Lamda value = 0.992, *F*_(1,200)_ = 1.53, *p* = 0.22, or for informant role, Wilks’ Lamda value = 0.997, *F*_(1,200)_ = 0.61, *p* = 0.43. Gender interacted with symptom type, Wilks’ Lamda = 0.981, *F*_(1,200)_ = 3.91, *p* = 0.049, but not with informant role, Wilks’ Lamda = 0.997, *F*_(1,200)_ = 0.52, *p* = 0.47. Between subject analysis revealed effect for gender, *F*_(1,200)_ = 10.04, *p* = 0.002. *Post hoc* analysis of the interaction effect yielded a mean difference of 2.99, *p* = 0.002. As depicted in Supplementary Figure S2, girls had more inattention problems than boys, but no gender difference was evident in the hyperactivity/impulsivity symptoms.

In order to examine whether gender differences exist in the agreement rates between teacher and parents rating of ADHD-related symptoms, we conducted a chi-square test. Gender differences were found in the rates of agreement on inattention problems *χ*^2^_(2, *N* = 204)_ = 12.08, *p* = 0.002 so that there were significantly more girls for whom both parent and teacher-reported inattention problems than boys who scored positive on both scales. In contrast, no gender differences were found in the agreement rates on hyperactivity/impulsivity symptoms. *χ*^2^_(2, *N* = 204)_ = 2.26, *p* = 0.88.

### Gender Differences in CPT Performance

To examine gender differences in MOXO-CPT performance, one-way repeated measures ANOVA was conducted, followed by posthoc analyses with Bonferroni correction for multiple comparisons. The eight test levels served as the within-subject factor and gender as the between-subject factor. The results are shown in Table 2.

**Table 2:** Gender differences in the four continuous performance test (CPT) performance indices.

CPT index	Test level	Boys (*n* = 129)		Girls (*n* = 75)		Gender differences^c^ (between-subject effect)
		Mean score		Mean score		
		M	SD	M	SD	
Attention	Base line	31.85	2.039	31.73	1.710	*F*_(1,193)_ = 0 0.38, *p* = 0.54
	Visual^a^	30.59	2.781	29.96	3.482
	Visual^b^	30.82	2.840	30.58	3.270
	Auditory^a^	30.63	2.878	30.29	3.238
	Auditory^b^	30.14	4.096	29.59	3.382
	Combined^a^	28.96	4.973	28.27	4.718
	Combined^b^	28.78	4.737	28.51	4.571
	No distractors	29.48	4.312	28.93	3.717
Timing	Base line	22.54	4.629	22.18	4.877	*F*_(1,193)_ = 1.93, *p* = 0.17
	Visual^a^	19.98	4.346	18.88	3.819
	Visual^b^	20.68	4.736	19.95	4.447
	Auditory^a^	21.62	4.733	20.79	4.670
	Auditory^b^	21.42	5.195	20.00	5.249
	Combined^a^	19.38	5.698	18.36	5.170
	Combined^b^	19.54	5.571	17.88	4.936
	No distractors	21.13	5.118	19.71	5.043
Hyperactivity	Base line	1.90	2.798	1.44	1.915	
	Visual^a^	3.76	4.940	2.63	2.176	*F*_(1,193)_ = 2.96, *p* = 0.09
	Visual^b^	5.90	8.341	4.41	5.838
	Auditory^a^	4.15	5.252	3.30	3.471
	Auditory^b^	5.31	7.731	4.53	7.288
	Combined^a^	6.02	9.115	5.23	10.161
	Combined^b^	7.25	11.287	4.78	5.197
	No distractors	4.48	5.759	4.29	6.315
Impulsivity	Base line	1.81	1.710	1.58	1.363	*F*_(1,193)_ = 4.63, *p* = 0.03
	Visual^a^	2.20	2.150	1.45	1.424
	Visual^b^	2.26	2.003	1.62	1.792
	Auditory^a^	2.37	2.148	1.88	1.779
	Auditory^b^	2.88	2.700	2.15	2.961
	Combined^a^	2.35	2.673	1.90	2.964
	Combined^b^	2.50	2.417	2.05	2.327
	No distractors	2.89	2.981	2.51	3.167	

*^a^Low distractibility (one distracting stimulus). ^b^High distractibility (two distracting stimuli). ^c^Based on two-way MANOVA with repeated measures. Note. In the Attention and Timing indices, higher scores mean better performance. In the Hyperactivity and Impulsivity indices, higher scores mean worse performance (increased hyperactive and impulsive responses)*.

Analyses of within subject effects on the Attention index revealed main effect of test level, Wilks’ Lamda = 0.738, *F*_(7,187)_ = 9.49, *p* < 0.001. Gender did not interact with test level, Wilks’ Lamda = 0.986, *F*_(7,187)_ = 0.39, *p* = 0.91. Between-subject analyses revealed no effect for gender, *F*_(1,193)_ = 0 0.38, *p* = 0.54.

Similar patterns were identified in the Timing and Hyperactivity indices. Analyses of within-subject effects in the Timing index revealed a main effect for test level, Wilks’ Lamda = 0.813, *F*_(8,187)_ = 6.14, *p* < 0.001. Gender did not interact with test level, Wilks’ Lamda = 0.969, *F*_(8,187)_ = 0.84, *p* = 0.55. Between-subject analyses revealed no effect for gender, *F*_(1,193)_ = 1.93, *p* = 0.17.

Analyses within subject effects on the Hyperactivity index revealed main effect for test level, Wilks’ Lamda = 0.723, *F*_(8,187)_ = 10.24, *p* < 0.001. Gender did not interact with test level, Wilks’ Lamda = 0.955, *F*_(8,187)_ = 1.27, *p* = 0.27. Between-subject analysis revealed no effect for gender, *F*_(1,193)_ = 2.96, *p* = 0.09.

Analyses of within-subject effects on the Impulsivity index revealed a main effect for test level, Wilks’ Lamda = 0.971, *F*_(1,193)_ = 5.67, *p* = 0.018. Gender did not interact with test level, Wilks’ Lamda = 0.968, *F*_(2,192)_ = 0.88, *p* = 0.52. However, between-subject analysis revealed a main effect of gender, *F*_(1,193)_ = 4.63, *p* = 0.03. *Post hoc* analyses with Bonferroni correction for multiple comparisons indicated that boys (*M* = 2.41, *SD* = 0.15) conducted more impulsive responses than girls (*M* = 1.88, SD = 0.20), regardless of test level (*p* = 0.03).

The effect of the test level that was observed in all CPT indices reflects the variation between levels in the presence, type, or load of distractors. These effects will be described in the next section.

Finally, we wished to examine whether boys and girls were differently affected by time on the task. Therefore, we compared boys and girls on the difference between the first and the last level of every CPT index, using two-way repeated-measures ANOVA. For these analyses, test level (first and last) and CPT index (Attention, Timing, Hyperactivity, and Impulsivity) were the within-subject factors and gender was the between-subject factor.

Within subject analysis revealed a main effect for CPT index, Wilks’ Lamda = 0.20, *F*_(3,191)_ = 250.77, *p* < 0.001 but not for the test level, Wilks’ Lamda = 0.998, *F*_(1,193)_ = 0.34, *p* = 0.56. Gender did not interact with CPT index, Wilks’ Lamda = 0.994, *F*_(3,191)_ = 0.41, *p* = 0.75 or with test level, Wilks’ Lamda = 0.996, *F*_(1,193)_ = 0.87, *p* = 0.35. The three-way interaction was not significant as well, Wilks’ Lamda = 0.991, *F*_(3,191)_ = 0.56, *p* = 0.64. The between subject analysis did not reveal a main effect for gender, *F*_(1,193)_ = 2.39, *p* = 0.12.

### Gender Differences in Distractibility

To study gender differences in distractibility level during CPT performance, a series of two-way repeated-measures ANOVAs were conducted. Separate analyses were conducted for each one of the four MOXO-CPT performance indices. For these analyses, distractor type (visual, auditory, or combined) and distractibility load (low or high distractibility) was the within-subject factors, and gender was the between-subject factor. The results are shown in Table 3.

**Table 3:** Gender differences in distractibility during CPT performance.

CPT index	Test level	Boys (*n* = 129)		Girls (*n* = 75)		Gender differences^c^ (between-subject effect)
		M	SD	M	SD	
Attention	Base line-visual^a^	1.26	2.45	1.77	2.80	*F*_(1,193)_ = 0.33, *p* = 0.57.
	Base line-visual^b^	1.03	2.57	1.16	2.70
	Base line-auditory^a^	1.23	2.53	1.44	2.45
	Base line-auditory^b^	1.72	3.91	2.14	2.72
	Base line-combined^a^	2.89	4.79	3.45	4.32
	Base line-combined^b^	3.07	4.42	3.22	4.04
Timing	Base line-visual^a^	2.55	2.91	3.30	3.09	*F*_(1,193)_ = 2.31, *p* = 0.13.
	Base line-visual^b^	1.85	3.73	2.23	3.24
	Base line-auditory^a^	0.92	3.67	1.38	3.59
	Base line-auditory^b^	1.11	4.43	2.18	4.20
	Base line-combined^a^	3.15	4.53	3.82	4.39
	Base line-combined^b^	3.00	5.03	4.30	4.53
Hyperactivity	Base line-visual^a^	−1.85	3.34	−1.19	2.49	*F*_(1,193)_ = 2.14, *p* = 0.15
	Base line-visual^b^	−4.00	7.03	−2.97	5.88
	Base line-auditory^a^	−2.24	4.09	−1.86	3.66
	Base line-auditory^b^	−3.41	6.60	−3.10	7.17
	Base line-combined^a^	−4.12	8.16	−3.79	10.16
	Base line-combined^b^	−5.35	10.51	−3.34	5.18
Impulsivity	Base line-visual^a^	−0.39	1.717	0.12	1.60	*F*_(1,193)_ = 2.40, *p* = 0.12.
	Base line-visual^b^	−0.45	1.99	−0.04	1.67
	Base line-auditory^a^	−0.56	1.78	−0.30	1.77
	Base line-auditory^b^	−1.07	2.61	−0.58	2.84
	Base line-combined^a^	−0.54	2.66	−0.33	2.83
	Base line-combined^b^	−0.68	2.56	−0.48	2.34	

*^a^Low distractibility (one distracting stimulus). ^b^High distractibility (two distracting stimuli). ^c^Based on two-way MANOVA with repeated measures. Note. In all MOXO-CPT indices, higher scores (in absolute value) mean increased distractibility*.

Analyses of within subject effects on the Attention index revealed main effects of distractor type, Wilks’ Lamda = 0.811, *F*_(2,192)_ = 22.38, *p* < 0.001, and distractibility load, Wilks’ Lamda = 0.975, *F*_(1,193)_ = 4.97, *p* = 0.03. Gender did not interact with distractor type, Wilks’ Lamda = 1.00, *F*_(2,192)_ = 0.04, *p* = 0.96, or with distractibility load, Wilks’ Lamda = 0.996, *F*_(1,193)_ = 0 0.77, *p* = 0.38. The three-way interaction between distractor type, distractibility load and gender was not significant, Wilks’ Lamda = 0.995, *F*_(2,192)_ = 0 0.51, *p* = 0.60. Between-subject analysis revealed no effect for gender, *F*_(1,193)_ = 0 0.33, *p* = 0.57.

*Post hoc* analysis with Bonferroni correction of the main effect of distractor type on the Attention index showed that participants were more distracted by the combination of visual and auditory distractors than by the presence of pure visual (mean difference = 1.84, *p* < 0.001) or pure auditory distractors (mean difference = 1.52, *p* < 0.001). *Post hoc* analysis of the effect of distractibility load did not reveal significant differences.

Analyses of within-subject effects on the Timing index revealed a main effect of distractor type, Wilks’ Lamda = 0.842, *F*_(2,192)_ = 17.97, *p* < 0.001, but not of distractibility load, Wilks’ Lamda = 0.995, *F*_(1,193)_ = 0.93, *p* = 0.34. Gender did not interact with distractor type, Wilks’ Lamda = 0.998, *F*_(2,192)_ = 0.19, *p* = 0.83, and not with distractibility load, Wilks’ Lamda = 0.996, *F*_(1,193)_ = 0 0.80, *p* = 0.37. The three-way interaction between distractor type, distractibility load, and gender was not significant, Wilks’ Lamda = 0.984, *F*_(2,192)_ = 1.51, *p* = 0.22. Between-subject analysis revealed no effect for gender, *F*_(1,193)_ = 2.31, *p* = 0.13. *Post hoc* analysis with Bonferroni correction of the main effect of distractor type on the Timing index showed that participants were more distracted by the combination of visual and auditory distractors than by pure visual (mean difference = 1.07, *p* < 0.001) or pure auditory (mean difference = 2.16, *p* < 0.001) distractors. Pure visual distractors were more distracting than pure auditory distractors (mean difference = 1.20, *p* < 0.001).

Analyses of within subject effects on the Hyperactivity index revealed main effect of distractibility load, Wilks’ Lamda = 0.910, *F*_(1,193)_ = 19.06, *p* < 0.001, but not for distractor type, Wilks’ Lamda = 0.990, *F*_(2,192)_ = 0.99, *p* = 0.38. Gender did not interact with distractor type, Wilks’ Lamda = 0.993, *F*_(2,192)_ = 0.71, *p* = 0.49 or with distractibility load, Wilks’ Lamda = 0.983, *F*_(1,193)_ = 3.29, *p* = 0.07. The three-way interaction between distractor type, distractibility load and gender was not significant, Wilks’ Lamda = 0.994, *F*_(2,192)_ = 0.54, *p* = 0.58. Between-subject analysis revealed no effect for gender, *F*_(1,193)_ = 2.14, *p* = 0.15. *Post hoc* analysis with Bonferroni correction of the main effect of distractibility load on the Hyperactivity index showed that CPT performance was worse when two distractors were simultaneously presented than when only one distractor was presented (mean difference = 1.10, *p* < 0.001).

Analyses of within-subject effects on the Impulsivity index revealed a main effect of distractibility load, Wilks’ Lamda = 0.971, *F*_(1,193)_ = 5.67, *p* = 0.018, but not of distractor type, Wilks’ Lamda, 0.994, *F*_(2,192)_ = 0.59, *p* = 0.56. Gender did not interact with distractor type, Wilks’ Lamda = 0.997, *F*_(2,192)_ = 0.34, *p* = 0.72 or with distractibility load, Wilks’ Lamda = 0.998, *F*_(1,193)_ = 0.23, *p* = 0.79. The three-way interaction between distractor type, distractibility load, and gender was not significant, Wilks’ Lamda = 0.997, *F*_(2,192)_ = 0.34, *p* = 0.72. The between-subject analysis did not reveal a main effect for gender, *F*_(1,193)_ = 2.40, *p* = 0.12. *Post hoc* analysis with Bonferroni correction of the main effect of distractor ability load on the Impulsivity index showed that CPT performance was worse when two distractors were simultaneously presented than when only one distractor was presented (mean difference = 0.21, *p* = 0.02).

## Discussion

This study systematically examined gender effects on ADHD manifestations in a clinic-referred sample of children with ADHD aged 6–17 years, as obtained through subjective and objective measures of ADHD symptoms. To reduce a reporter’s bias, the current study used the CPT as an objective laboratory-based measure of ADHD symptoms. To the best of our knowledge, this is the first study that focused on gender differences in distractibility in children with ADHD.

Examination of gender differences in parent and teacher reports on ADHD-related symptoms, according to the Conners rating scales, showed that the level of inattention symptoms was higher among referred girls. However, boys and girls were equally impaired in terms of impulsivity and hyperactivity. A similar pattern emerged in the TRF, where teachers reported more inattention problems for girls, but higher levels of depression, anxiety, and rule-breaking behaviors for boys. Consistent with previous studies, these findings suggest that clinically diagnosed males and females showed similar symptom severity except for higher inattention scores in females (Biederman et al., 2002; Biederman and Faraone, 2004; Graetz et al., 2005). In addition, we found that teachers, but not parents, were likely to identify boys as having more psychiatric internalizing (anxiety/depression) and externalizing (rule-breaking) co-occurring symptoms. These findings differ from previous studies, which found more anxious/depressed symptoms in girls than in boys (Quinn, 2008; Liu et al., 2011). Probably, teachers and parents capture different aspects of depression and anxiety (e.g., fear of novel experiences vs. school-related anxiety; Geiser, 2009; Grigorenko et al., 2010). For instance, teachers may be more likely than parents to identify anxiety and depression because ADHD-related social and academic difficulties are more prominent in the school environment (Biederman et al., 1995). Alternatively, it is possible that boys’ externalizing symptoms were associated with elevated levels of emotional lability and dysregulation (Martel and Nigg, 2006; Seymour et al., 2014) and therefore were pronounced as anxiety and depression.

While teachers’ and parents’ ADHD rating scales demonstrated gender differences in the inattention domain, a different pattern of gender differences emerged in the CPT data. Similar to previous studies (McGee et al., 2000; Seidman et al., 2005; Miranda et al., 2012), our results showed that boys conducted more impulsive responses than girls, regardless the presence of distracting stimuli, the type of distractors (visual, auditory or combined) or their load (one or two distracting stimuli at a time). Importantly, gender did not interact with distractors type or with their load, indicating that the effect of environmental distractors on CPT performance did not significantly differ between boys and girls. Our results suggest a possible dissociation between gender and the method of ADHD assessment; while girls with ADHD (but not boys) showed increased inattention symptoms according to teacher and parent report scales, boys (but not girls) showed increased impulsivity according to CPT performance indices. These results indicate that distinct ADHD-related deficits might be evaluated by different assessment methods and might also be differently patterned in males and females.

Nonspecific associations between CPT performance and behavioral measures of ADHD have been well documented in the ADHD literature (Reh et al., 2015; Willard et al., 2016). CPT performance was poorly to moderately correlated with parent and teacher ratings and was often inconsistent with subtypes of ADHD diagnosis (Barkley, 1991; Edwards et al., 2007). Several interpretations were offered for these findings. First, it has been suggested that CPT performance and behavioral measures of ADHD may not converge due to the limited utility and ecological validity of both assessment methods. Some authors have questioned the validity of parent and teacher reports, given their vulnerability to clinician and informant biases (Edwards et al., 2007), reduced reliability for monitoring symptoms over time (Rabiner et al., 2010), and the influences of ethnicity, gender and socioeconomic status on ADHD symptom ratings (Slobodin and Masalha, in press).

On the other hand, several authors have questioned the utility and the ecological validity of the CPT in the diagnosis of ADHD, as it provides only a brief snapshot of a child’s attentional capacity in a controlled environment (Barkley, 1991; Netson et al., 2011). A second possible explanation for the low convergent validity of the CPT with other behavioral measures of ADHD is that the magnitude of response achieved on CPT differs from that perceived by parents and teachers. For example, McGee et al. (2000) found that in a sample of clinic-referred children, the CPT was sensitive to behavior ratings only at the highest levels of the behavioral disturbance. The limited sensitivity of the CPT to ADHD-related deficits might be attributed to insufficient cognitive demands of the test (Mahone et al., 2001; Berlin et al., 2004), leading to a ceiling effect in CPT performance (Lasee and Choi, 2013). Third, laboratory and behavioral measures of ADHD may be tapping into qualitatively different aspects of behavior. Behavior ratings are based on the accumulation of behavior during extended periods that occur in real-life situations. CPT, on the other hand, measures behavior in a particular moment in a laboratory setting (Barkley, 1991). Likewise, the CPT may not capture some aspects of ADHD that are perceived by behavioral ratings, such as hyperactivity (Reh et al., 2015). There is evidence that when a measurement of activity was combined with the CPT, its convergence validity with teacher ratings significantly improved as well its ability to distinguish between ADHD and non-ADHD cases (Reh et al., 2015). Finally, the low correspondence between ratings of behavior and constructs measured by CPT may be related to the fact the CPT fails to demonstrate symptom domain specificity (Epstein et al., 2006; Netson et al., 2011). In a large epidemiological study, Epstein et al. (2006) found that unexpectedly, omission errors were associated with hyperactivity symptoms (and not with inattention symptoms), whereas commission errors were related to impulsivity symptoms, hyperactivity, and inattention symptoms. Likewise, a recent study demonstrated that the levels of hyperactivity or impulsivity during CPT performance might be associated with basic attentional rather than inhibition processes (Vogt et al., 2018). It has been suggested that excessive motoric activity, such as fidgeting during a cognitive performance, reflects efforts to modulate attention and alertness (Hartanto et al., 2016). Thus, children with predominantly inattention problems may demonstrate higher levels of activity during CPT performance than children with predominantly hyperactivity/impulsivity deficits.

The above findings suggest that the limited, nonspecific associations between CPT performance and behavioral measures of ADHD are multi-factorial and may reflect the psychometric properties of both assessment methods as well as the underlying ADHD-related deficits. Given the lack of research on the correlations between gender, ADHD symptomatology, and CPT performance (Sims and Lonigan, 2012), further investigation is needed. Such an examination might assist clinicians in interpreting the similarities and differences in these two sources of data and how they are affected by gender.

A question remains as to whether and how gender differences in CPT performance may vary as a function of the paradigm’s requirements. In their meta-analytic study of gender differences in CPT performance, Hasson and Fine (2012) indicated that the type of the included CPTs (i.e., Conners CPT, AX-CPT, and an auditory CPT) did not significantly contribute to their overall findings. However, it was impossible to determine how each CPT version contributed to the overall observed gender differences due to the heterogeneity of CPT paradigms and the different weights imposed on each study. The MOXO-CPT has numerous unique aspects that might have affected our results. First, the MOXO-CPT may pose a higher distractibility load than other CPT paradigms. In most CPTs that involve distracting stimuli, auditory distractors served as background noise while children performed another cognitive task (Abikoff et al., 1996; Pelham et al., 2011). In contrast, distractors in the MOXO-CPT vary in their type, length of presentation and location on the screen. This mode of presentation did not allow adjustment or de-sensitization to the distractors, thus maintaining high distractibility load throughout the test.

Further, while some studies have used neutral stimuli (neutral tone/letter) as distractors (Gordon and Mettelman, 1987; Uno et al., 2006; van Mourik et al., 2007), the MOXO-CPT incorporated ecologically valid stimuli that are typically found in the child’s or adolescent’s everyday environment. Because patients with ADHD have more difficulties in filtering meaningful distractors than neutral ones (Blakeman, 2000; López-Martín et al., 2013), it is possible that this feature of the test further contributed to its high distractibility load. These increased attention demands may have led to a floor effect in CPT performance of both males and females, thus hindering our ability to observe gender differences on various CPT indices, such as sustained attention and timing. A second aspect of the MOXO-CPT that should be considered when interpreting our results is that the test distinguishes between commission errors associated with impulsivity and hyperactive responses associated with increased activity level. While previous CPT studies consistently showed higher rates of commission responses among boys than girls (Hasson and Fine, 2012), they could not indicate whether these uninhibited responses were associated with impulsivity or with increased activity level (Pettersson et al., 2018). The MOXO-CPT may offer a more nuanced observation of gender differences in these two ADHD-related symptoms, suggesting that boys may be more impulsive but not more active than girls. Finally, the MOXO-CPT may differ from other CPTs in the level of attentional demands over time. Previous studies (Bioulac et al., 2012; Erdodi and Lajiness-O’Neil, 2013) showed decreased attention in children and adolescents with ADHD as the task progressed, probably due to degraded executive functions and/or motivational resources (Baumeister et al., 2007; Inzlicht and Schmeichel, 2012; Dekkers et al., 2017). However, we did not find any differences between participants’ CPT scores in the first and the last level of the test, regardless of their gender or the CPT index. One possible explanation for the diversity of our findings may be related to the cognitive complexity of the MOXO-CPT paradigm. Previous research has associated the decreased cognitive performance over time in children and adolescents with ADHD with the increased complexity of the task (Tucha et al., 2009, 2017; Huang-Pollock et al., 2012). For example, in a study with children diagnosed with ADHD, Bioulac et al. (2012) found a deterioration of performances over time in a virtual classroom task but not in the CPT. They suggested that virtual classroom task involved more complex cognitive mechanisms than the CPT, which imposed only minimal working memory load. Although the MOXO-CPT required multiple attentional resources (various inter-stimuli intervals, high burden of distractors, the use of ecologically valid distractors, and various locations and types of stimuli), the first and the last level of the test were always free of distractors and may, therefore, required fewer cognitive demands compared to the other test levels. The fact that order effects confounded with the effects of test conditions (various distractor type and load) may explain why the current study failed to identify time-on-task effects in both genders. Nevertheless, the interaction between gender and time-on-task effect on CPT performance should be further addressed in future studies.

Several limitations of this study should be considered. The first limitation is associated with the limited generalizability of the study, due to relative ethnic and geographic homogeneity of the sample, its limited size, and the fact that all children were recruited from a single neuro-pediatric clinic. Given that sustained attention is related to socio-cultural factors and even to gender equality in the country (Riley et al., 2016), our sampling method limits our ability to generalize our findings to populations with greater cultural diversity. Another factor that may limit the generalization of our findings is the fact that we included only Israeli patients. Israel is characterized by a dramatic rise in ADHD referral and diagnosis rates, mainly due to increased community knowledge about symptomatology and the benefits of treatment (Davidovitch et al., 2017). The current sample included relatively mild cases of ADHD, thus limiting the generalization of our findings to countries with lower referral rates. Another limitation of the study is our inability to study gender differences separately for children and adolescents due to our limited sample size. Indices of CPT performance, including the level of distractibility, were previously associated with age (Berger et al., 2013; Slobodin et al., 2015). Therefore, it will be worthwhile to study gender effects on developmental trajectories of ADHD. Finally, it should be noted that compared to other well-established CPTs, the MOXO-CPT is a relatively novel tool with more limited empirical support. Further investigation is needed to provide insight into its psychometrical properties in the ADHD evaluation process.

Our results may offer further insight into the various effects of gender on rating and objective measures of ADHD. The current study suggests that attention deficits according to parents’ and teacher’s rating scales were stronger predictors of ADHD referral among girls, whereas externalization behavior were stronger predictors of ADHD referral among boys. However, when assessed on objective measures of ADHD, boys were more impulsive than girls, but no other gender differences were observed. The fact that boys and girls did not differ on most CPT outcomes, including attention performance, timing, motor activity, distractibility, and time-on-task, may indicate that the severity of their ADHD symptoms is overall similar. These results are in line with previous research in clinically ascertained samples, showing similar levels of ADHD symptoms in boys and girls in ADHD rating scales with the exception of inattention for which females had higher ratings (Gershon, 2002; Mowlem et al., 2019).

Although the utility of the CPT as a stand-alone diagnostic tool for ADHD is currently limited (Berger et al., 2017; Tallberg et al., 2019), it may add valuable information about ADHD-related deficits to support clinical diagnosis. Using an objective laboratory-based measure of ADHD may be especially important among girls, who, due to clinician and informant biases, tend to be underdiagnosed and undertreated for ADHD (Coles et al., 2012). Our findings may encourage clinicians and researchers to consider using gender-specific norms and guidelines when assessing symptoms of ADHD (Hasson and Fine, 2012).

## Conclusions

The present study provides insight into gender differences in ADHD symptoms using subjective and objective measures of ADHD. It demonstrated that parents and teachers were more likely to identify girls as having inattention problems than boys. Teachers were more likely to identify rule-breaking and anxiety/depression symptoms in boys than in girls. CPT analysis revealed higher impulsivity among boys. Gender did not interact with distractors type or load to affect CPT performance, suggesting that deficits in inhibition control and self-regulation might be considered a key aspect of ADHD in both boys and girls (Barkley, 1997, 1999). These findings highlight the need to include multiple sources of information and methods of assessment to reduce the gender gap in referred children.

## Data Availability Statement

The datasets generated for this study are available on request to the corresponding author.

## Ethics Statement

The studies involving human participants were reviewed and approved by Ethics Committee of Maccabi Health Services. Written informed consent to participate in this study was provided by the participants’ legal guardian/next of kin.

## Author Contributions

MD recruited the patients and performed clinical evaluations. OS and MD contributed equally to the development of study design, data analysis, integration of findings, and writing the manuscript.

## Conflict of Interest

OS has previously served on the scientific advisory board of NeuroTech Solutions Limited. The remaining author declares that the research was conducted in the absence of any commercial or financial relationships that could be construed as a potential conflict of interest.

## Footnotes

^1^The term “MOXO” derives from the world of Japanese martial arts and means a “moment of lucidity.” It refers to the moments preceding the fight, when the warrior clears his mind from distracting, unwanted thoughts, and feelings.

## References

[B1] Abikoff H. Courtney M. E. Szeibel P. J. Koplewicz H. S. (1996). The effects of auditory stimulation on the arithmetic performance of children with ADHD and nondisabled children. J. Learn. Disabil. 29, 238–246. 10.1177/002221949602900302 8732885

[B2] Achenbach T. M. Rescorla L. A. (2001). Manual for the ASEBA School-Age Forms and Profiles: An Integrated System of Multi-Informant Assessment. Burlington, NJ: University of Vermont, Research Center for Children, Youth and Families.

[B3] American Psychiatric Association (2013). Diagnostic and Statistical Manual of Mental Disorders. 5th Edn. Washington, DC: American Psychiatric Association.

[B4] Arnold L. E. (1996). Sex differences in ADHD: conference summary. J. Abnorm. Child Psychol. 24, 555–569. 10.1007/bf01670100 8956084

[B5] Barkley R. A. (1991). The ecological validity of laboratory and analogue assessment methods of ADHD symptoms. J. Abnorm. Child Psychol. 19, 149–178. 10.1007/bf00909976 2056161

[B6] Barkley R. A. (1997). ADHD and the Nature of Self-Control. New York, NY: Guilford Press.

[B7] Barkley R. A. (1999). Response inhibition in attention deficit hyperactivity disorder. Ment. Retard. Dev. Dis. Res. Rev. 5, 177–184. 10.1002/(SICI)1098-2779(1999)5:3<177::AID-MRDD3>3.0.CO;2-G

[B8] Barkley R. A. (2015). Attention-Deficit Hyperactivity Disorder: A Handbook for Diagnosis and Treatment. 4th Edn. New York, NY: The Guilford Press.

[B9] Baumeister R. F. Vohs K. D. Tice D. M. (2007). The strength model of self-control. Cur. Dir. Psychol. Sci. 16, 351–355. 10.1111/j.1467-8721.2007.00534.x

[B10] Berger I. Cassuto H. (2014). The effect of environmental distractors incorporation into a CPT on sustained attention and ADHD diagnosis among adolescents. J. Neurosci. Methods 222, 62–68. 10.1016/j.jneumeth.2013.10.012 24211249

[B11] Berger I. Goldzweig G. (2010). Objective measures of attention-deficit/hyperactivity disorder: a pilot study. Isr. Med. Assoc. J. 12, 531–535. 10.1016/j.jneumeth.2013.10.012 21287795

[B13] Berger I. Slobodin O. Aboud M. Melamed J. Cassuto H. (2013). Maturational delay in ADHD: evidence from CPT. Front. Hum. Neurosci. 7:691. 10.3389/fnhum.2013.00691 24298243 PMC3829464

[B12] Berger I. Slobodin O. Cassuto H. (2017). Usefulness and validity of CPT in the diagnosis of ADHD children. Arch. Clin. Neuropsychol. 32, 81–93. 10.1093/arclin/acw101 28122767

[B14] Berlin L. Bohlin G. Nyberg L. Janols L. O. (2004). How well do measures of inhibition and other executive functions discriminate between children with ADHD and controls? Child Neuropsychol. 10, 1–13. 10.1076/chin.10.1.1.26243 14977511

[B15] Biederman J. Faraone S. Mick E. Lelon E. (1995). Psychiatric comorbidity among referred juveniles with major depression: fact or artifact? J. Am. Acad. Child Adolesc. Psychiatry 34, 579–590. 10.1097/00004583-199505000-00010 7775353

[B18] Biederman J. Faraone S. V. (2004). The massachusetts general hospital studies of gender influences on attention-deficit/hyperactivity disorder in youth and relatives. Psychiatr. Clin. North Am. 72, 225–232. 10.1016/j.psc.2003.12.004 15063995

[B19] Biederman J. Kwon A. Aleardi M. Chouinard V. A. Marino T. Cole H.. (2005). Absence of gender effects on attention deficit hyperactivity disorder: findings in nonreferred subjects. Am. J. Psychiatry 162, 1083–1089. 10.1176/appi.ajp.162.6.1083 15930056

[B16] Biederman J. Mick E. Faraone S. V. Braaten E. Doyle A. Spencer T.. (2002). Influence of gender on attention deficit hyperactivity disorder in children referred to a psychiatric clinic. Am. J. Psychiatry 159, 36–42. 10.1176/appi.ajp.159.1.36 11772687

[B17] Biederman J. Petty C. R. Monuteaux M. C. Fried R. Byrne D. Mirto T.. (2010). Adult psychiatric outcomes of girls with attention deficit hyperactivity disorder: 11-year follow-up in a longitudinal case-control study. Am. J. Psychiatry 167, 409–417. 10.1176/appi.ajp.2009.09050736 20080984

[B20] Bioulac S. Lallemand S. Rizzo A. Philip P. Fabrigoule C. Bouvard M. P. (2012). Impact of time on task on ADHD patient’s performances in a virtual classroom. Eur. J. Psychiatry 16, 514–521. 10.1016/j.ejpn.2012.01.006 22269913

[B21] Blakeman R. S. (2000). ADHD and distractibility: the role of distractor appeal. Diss. Abstr. Int. B. Sci. Eng. 61:517.

[B22] Bruchmüller K. Margraf J. Schneider S. (2012). Is ADHD diagnosed in accord with diagnostic criteria? Overdiagnosis and influence of client gender on diagnosis. J. Consult. Clin. Psychol. 80, 128–138. 10.1037/a0026582 22201328

[B23] Cohen J. (1992). A power primer. Psychol. Bull. 112, 155–159. 10.1037/0033-2909.112.1.155 19565683

[B24] Coles E. K. Slavec J. Bernstein M. Baroni E. (2012). Exploring the gender gap in referrals for children with ADHD and other disruptive behavior disorders. J. Atten. Disord. 16, 101–108. 10.1177/1087054710381481 20837979

[B25] Conners C. K. (2008). Conners. 3rd Edn. Toronto, Ontario: Multi-Health Systems Inc.

[B26] Davidovitch M. Koren G. Fund N. Shrem M. Porath A. (2017). Challenges in defining the rates of ADHD diagnosis and treatment: trends over the last decade. BMC Pediatr. 29:218. 10.1186/s12887-017-0971-0 29284437 PMC5747128

[B27] Dekkers T. Agelink van Rentergem J. A. Koole A. van den Wildenberg W. P. M. Popma A. Bexkens A.. (2017). Time-on-task effects in children with and without ADHD: depletion of executive resources or depletion of motivation? Eur. Child Adolesc. Psychiatry 26, 1471–1481. 10.1007/s00787-017-1006-y 28536846 PMC5701950

[B28] Diamantopoulou S. Rydell A. M. Thorell L. B. Bohlin G. (2007). Impact of executive functioning and symptoms of attention deficit hyperactivity disorder on children’s peer relations and school performance. Dev. Neuropsychol. 31, 521–542. 10.1080/87565640701360981 17650992

[B29] Edwards M. C. Gardner E. S. Chelonis J. J. Schulz E. G. Flake R. A. Diaz P. F. (2007). Estimates of the validity and utility of the Conner’s CPT in the assessment of inattentive and/or hyperactive impulsive behaviors in children. J. Abnorm. Child Psychol. 35, 393–404. 10.1007/s10802-007-9098-3 17295064

[B30] Epstein J. N. Conners C. K. Hervey A. S. Tonev S. T. Arnold L. E. Abikoff H.. (2006). Assessing medication effects in the MTA study using neuropsychological outcomes. J. Child Psychol. Psychiatry 47, 446–456. 10.1111/j.1469-7610.2005.01469.x 16671928

[B31] Erdodi L. A. Lajiness-O’Neil R. (2013). Time-related changes in Conners’ CPT-II scores: a replication study. Appl. Neuropsychol. Adult 21, 41–50. 10.1080/09084282.2012.724036 24826495

[B32] Gaub M. Carlson C. I. (1997). Gender differences in ADHD: a meta-analysis and critical review. J. Am. Acad. Child Adolesc. Psychiatry 36, 1036–1045. 10.1097/00004583-199708000-00011 9256583

[B33] Geiser C. (2009). Multitrait-Multimethod-Multioccasion Modeling. München, Germany: AVM.

[B34] Gershon J. (2002). A meta-analytic review of gender differences in ADHD. J. Atten. Disord. 5, 143–154. 10.1177/108705470200500302 11911007

[B35] Glass C. S. Wegar K. (2000). Teacher perceptions of the incidence and management of attention deficit hyperactivity disorder. Education 121, 412–420.

[B36] Gordon M. Mettelman B. B. (1987). Technical Guide to the Gordon Diagnostic System. Syracuse, NY: Gordon Systems.

[B37] Graetz B. W. Sawyer M. G. Baghurst P. (2005). Gender differences among children with DSM-IV ADHD in Australia. J. Am. Acad. Child Adolesc. Psychiatry 44, 159–168. 10.1097/00004583-200502000-00008 15689729

[B38] Grigorenko E. L. Geiser C. Slobodskaya H. R. Francis D. J. (2010). Cross-informant symptoms from CBCL, TRF, and YSR: trait and method variance in a normative sample of Russian youths. Psychol. Assess. 22, 893–911. 10.1037/a0020703 21133549 PMC4315166

[B39] Gudjonsson G. H. Sigurdsson J. F. Sigfusdottir I. D. Young S. (2014). A national epidemiological study of offending and its relationship with ADHD symptoms and associated risk factors. J. Atten. Disord. 18, 3–13. 10.1177/1087054712437584 22522573

[B40] Hartanto T. A. Krafft C. E. Iosif A. M. Schweitzer J. B. (2016). A trial-by-trial analysis reveals more intense physical activity is associated with better cognitive control performance in attention-deficit/hyperactivity disorder. Child Neuropsychology 22, 618–626. 10.1080/09297049.2015.1044511 26059476 PMC4675699

[B41] Hasson R. Fine J. G. (2012). Gender differences among children with ADHD on continuous performance tests: a meta-analytic review. J. Atten. Disord. 16, 190–198. 10.1177/1087054711427398 22290696

[B42] Hezi L. (2010). Criteria for Diagnosing ADHD in Children, Adolescents and Adults. Israeli Ministry of Health Available online at: http://www.health.gov.il/hozer/mr40_2010.pdf. Accessed November 20, 2019.

[B44] Hinshaw S. P. Owens E. B. Sami N. Fargeon S. (2006). Prospective follow-up of girls with attention-deficit/hyperactivity disorder into adolescence: evidence for continuing cross-domain impairment. J. Consult. Clin. Psychol. 74, 489–499. 10.1037/0022-006x.74.3.489 16822106

[B43] Hinshaw S. P. Owens E. B. Zalecki C. Huggins S. P. Montenegro-Nevado A. J. Schrodek E.. (2012). Prospective follow-up of girls with attention-deficit/hyperactivity disorder into early adulthood: continuing impairment includes elevated risk for suicide attempts and self-injury. J. Consult. Clin. Psychol. 80, 1041–1051. 10.1037/a0029451 22889337 PMC3543865

[B45] Huang-Pollock C. L. Karalunas S. L. Tam H. Moore A. N. (2012). Evaluating vigilance deficits in ADHD: a meta-analysis of CPT performance. J. Abnorm. Psychol. 121, 360–371. 10.1037/a0027205 22428793 PMC3664643

[B46] Inzlicht M. Schmeichel B. J. (2012). What is ego depletion? Toward a mechanistic revision of the resource model of self-control. Perspect. Psychol. Sci. 7, 450–463. 10.1177/1745691612454134 26168503

[B47] Israeli Central Bureau of Statistics (2017). Characterization and Classification of Geographical Units by the Socio-Economic Level of the Population, 2013. Available online at: https://www.cbs.gov.il/en/publications/Pages/2017/Characterization-and-Classification-of-Geographical-Units-by-the-Socio-Economic-Level-of-the-Population-2013.aspx. Accessed November 20, 2019.

[B48] Lambert M. C. Rowan G. T. Lyubansky M. Russ C. M. (2002). Do problems of clinic-referred African-American children overlap with the child behavior checklist? J. Child. Fam. Stud. 11, 271–285. 10.1023/A:101681600

[B49] Lasee M. J. Choi H.-S. (2013). Evidence of Reliability and validity for a children’s auditory continuous performance test. SAGE Open 3:215824401351182 10.1177/2158244013511828

[B50] Liu J. Cheng H. Leung P. W. (2011). The application of the preschool Child Behavior Checklist and the caregiver-teacher report form to Mainland Chinese children: syndrome structure, gender differences, country effects, and inter-informant agreement. J. Abnorm. Child Psychol. 39, 251–264. 10.1007/s10802-010-9452-8 20821258 PMC3042499

[B51] López-Martín S. Albert J. Fernández-Jaén A. Carretié L. (2013). Emotional distraction in boys with ADHD: neural and behavioral correlates. Brain Cogn. 83, 10–20. 10.1016/j.bandc.2013.06.004 23867737

[B52] Mahone M. E. Pillion J. P. Hiemenz J. R. (2001). Initial development of an auditory continuous performance test for preschoolers. J. Atten. Disord. 5, 93–106. 10.1177/108705470100500203

[B53] Martel M. M. Nigg J. T. (2006). Child ADHD and personality/temperament traits of reactive and effortful control, resiliency, and emotionality. J. Child. Psychol. Psychiatry 47, 1175–1183. 10.1111/j.1469-7610.2006.01629.x 17076757

[B54] McGee R. A. Clark S. E. Symons D. K. (2000). Does the conners’ continuous performance test aid in ADHD diagnosis? J. Abnorm. Child Psychol. 28, 415–424. 10.1023/a:1005127504982 11100916

[B55] Meyer B. J. Stevenson J. Sonuga-Barke E. J. S. (2017). Sex differences in the meaning of parent and teacher ratings of ADHD behaviors: an observational study. J. Atten. Disord. [Epub ahead of print]. 10.1177/1087054717723988 28800718

[B56] Miranda M. C. Rivero T. S. Amodeo Bueno O. F. (2012). Effects of age and gender on performance on Conners’ Continuous Performance Test in Brazilian adolescents. Psychol. Neurosci. 6, 73–78. 10.3922/j.psns.2013.1.11

[B57] Mowlem F. D. Rosenqvist M. A. Martin J. Lichtenstein P. Asherson P. Larsson H. (2019). Sex differences in predicting ADHD clinical diagnosis and pharmacological treatment. Eur. Child Adolesc. Psychiatry 28, 481–489. 10.1007/s00787-018-1211-3 30097723 PMC6445815

[B58] Nadeau K. Quinn P. (2002). “Rethinking the DSM-I,” in Understanding Women with ADHD, eds Nadeau K. Quinn P. (Silver Spring: Advantage Books), 2–23.

[B59] National Institute of Mental Health (2012). Attention Deficit Hyperactivity Disorder. Available online at: http://www.nimh.nih.gov/health/publications/attention-deficit-hyperactivity-disorder/complete-index.shtml. Accessed June 03, 2019.

[B60] Netson K. L. Conklin H. M. Ashford J. M. Kahalley L. S. Wu S. Xiong X. (2011). Parent and teacher ratings of attention during a year-long methylphenidate trial in children treated for cancer. J. Pediatr. Psychol. 36, 438–450. 10.1093/jpepsy/jsq102 21097489 PMC3115681

[B61] Nøvik T. S. Hervas A. Ralston S. J. Dalsgaard S. Rodrigues Pereira R. Lorenzo M. J. (2006). Influence of gender on attention-deficit/hyperactivity disorder in Europe—ADORE. Eur. Child Adolesc. Psychiatry 15, 15–24. 10.1007/s00787-006-1003-z 17177011

[B62] Ohan J. L. Visser T. A. (2009). Why is there a gender gap in children presenting for attention-deficit/hyperactivity disorder services? J. Clin. Child Adolesc. Psychol. 38, 650–660. 10.1080/15374410903103627 20183650

[B63] Papageorgiou V. Kalyva E. Dafoulis V. Vostanis P. (2008). Differences in parents’ and teachers’ ratings of ADHD symptoms and other mental health problems. Eur. J. Psychiatry 22, 200–210. 10.4321/s0213-61632008000400003

[B64] Pelham W. E. Jr. Waschbusch D. A. Hoza B. Gnagy E. M. Greiner A. R. Sams S. E.. (2011). Music and video as distractors for boys with ADHD in the classroom: comparison with controls, individual differences, and medication effects. J. Abnorm. Child Psychol. 39, 1085–1098. 10.1007/s10802-011-9529-z 21695447

[B65] Pettersson R. Söderström S. Nilsson K. W. (2018). Diagnosing ADHD in adults: an examination of the discriminative validity of neuropsychological tests and diagnostic assessment instruments. J. Atten. Disord. 22, 1019–1031. 10.1177/1087054715618788 26681530

[B66] Psychtech Ltd (2005). Translation of the Achenbach System for Evidence-Based Assessment. Jerusalem: PsychTech Ltd.

[B67] Psychtech Ltd (2012). Translation of the Conners 3AI Rating Scales. Jerusalem: PsychTech Ltd.

[B68] Quinn P. O. (2008). Review Attention-deficit/hyperactivity disorder and its comorbidities in women and girls: an evolving picture. Curr. Psychiatry Rep. 10, 419–423. 10.1007/s11920-008-0067-5 18803916

[B69] Quinn P. O. (2010). 100 Questions and Answers About Attention Deficit Hyperactivity Disorder (ADHD) in Women and Girls. Burlington, MA: Jones and Bartlett Learning.

[B70] Quinn P. O. Madhoo M. (2014). A review of attention-deficit/hyperactivity disorder in women and girls: uncovering this hidden diagnosis. Prim. Care Companion CNS Disord. 16:PCC.13r01596. 10.4088/pcc.13r01596 25317366 PMC4195638

[B71] Rabiner D. L. Murray D. W. Skinner A. T. Malone P. S. (2010). A randomized trial of two promising computer-based interventions for students with attention difficulties. J. Abnorm. Child Psychol. 38, 131–142. 10.1007/s10802-009-9353-x 19697119

[B72] Ramtekkar U. Reiersen A. Todorov A. Todd R. (2010). Sex and age differences in attention-deficit/hyperactivity disorder symptoms and diagnoses: implications for DSM-V and ICD-11. J. Am. Acad. Child Adolesc. Psychiatry 49, 217.e3–228.e3. 10.1016/j.jaac.2009.11.011 20410711 PMC3101894

[B73] Reh V. Schmidt M. Lam L. Schimmelmann B. G. Hebebrand J. Rief W.. (2015). Assessment of core ADHD symptoms using the QbTest. J. Atten. Disord. 19, 1034–1045. 10.1177/1087054712472981 23382579

[B74] Riley E. Okabe H. Germine H. Wilmer J. Esterman M. DeGutis J. (2016). Gender differences in sustained attentional control relate to gender inequality across countries. PLoS One 11:e0165100. 10.1371/journal.pone.0165100 27802294 PMC5089545

[B75] Rousseau C. Measham T. Bathiche-Suidan M. (2008). DSM-IV, culture and child psychiatry. J. Can. Acad. Child Adolesc. Psychiatry 17, 69–75. 18516309 PMC2387108

[B77] Rucklidge J. J. (2008). Gender differences in ADHD: implications for psychosocial treatments. Expert Rev. Neurother. 8, 643–655. 10.1586/14737175.8.4.643 18416665

[B76] Rucklidge J. J. (2010). Gender differences in attention-deficit/hyperactivity disorder. Psychiatr. Clin. North Am. 33, 357–373. 10.1016/j.psc.2010.01.006 20385342

[B78] Sassi R. B. (2010). Attention-deficit hyperactivity disorder and gender. Arch. Womens Ment. Health 13, 29–31. 10.1007/s00737-009-0121-2 20127451

[B79] Sciutto M. J. Nolfi C. J. Bluhm C. (2004). Effects of child gender and symptom type on referrals for ADHD by elementary school teachers. J. Emot. Behav. Disord. 12, 247–253. 10.1177/10634266040120040501

[B81] Seidman L. J. Biederman J. Monuteaux M. Doyle A. E. Faraone S. V. (2001). Learning disabilities and executive dysfunction in boys with attention-deficit/hyperactivity disorder. Neuropsychology 15, 544–556. 10.1037/0894-4105.15.4.544 11761044

[B80] Seidman L. Biederman J. Monuteaux M. Valera E. Doyle A. Faraone S. (2005). Impact of gender and age on executive functioning: do girls and boys with and without attention deficit hyperactivity disorder differ neuropsychologically in preteen and teenage years? Dev. Neuropsychol. 27, 79–105. 10.1207/s15326942dn2701_4 15737943

[B82] Seymour K. E. Chronis-Tuscano A. Iwamoto D. K. Kurdziel G. Macpherson L. (2014). Emotion regulation mediates the association between ADHD and depressive symptoms in a community sample of youth. J. Abnorm. Child Psychol. 42, 611–621. 10.1007/s10802-013-9799-8 24221724 PMC4207628

[B83] Shahaf G. Nitzan U. Erez G. Meddelovic S. Bloch Y. (2018). Monitoring attention in ADHD with an easy-to-use electrophysiological index. Front. Hum. Neurosci. 12:32. 10.3389/fnhum.2018.00032 29449806 PMC5799268

[B84] Sims D. S. Lonigan C. J. (2012). Multi-method assessment of ADHD characteristics in preschool children: relations between measures. Early Child. Res. Q. 27, 329–337. 10.1016/j.ecresq.2011.08.004 22518069 PMC3327380

[B85] Skogli E. W. Teicher M. H. Anderen P. N. Hovik K. T. Øie M. (2013). ADHD in girls and boys—gender differences in co-existing symptoms and executive function measures. BMC Psychiatry 13:298. 10.1186/1471-244x-13-298 24206839 PMC3827008

[B87] Slobodin O. Cassuto H. Berger I. (2015). Age-related changes in distractibility: developmental trajectory of sustained attention in ADHD. J. Atten. Disord. 22, 1333–1343. 10.1177/1087054715575066 25791438

[B86] Slobodin O. Masalha R. (in press). Challenges in ADHD care for ethnic minority children: a review of the current literature. Transcult. Psychiatry 10.1177/1363461520902885 32233772

[B88] Tallberg P. Råstam M. Wenhov L. Eliasson G. Gustafsson P. (2019). Incremental clinical utility of continuous performance tests in childhood ADHD—an evidence-based assessment approach. Scand. J. Psychol. 60, 26–35. 10.1111/sjop.12499 30452083 PMC7379623

[B89] Thomas R. Sanders S. Doust J. Beller E. M. Glasziou P. (2015). Prevalence of attention-deficit/hyperactivity disorder: a systematic review and meta-analysis. Pediatrics 135, e994–e1001. 10.1542/peds.2014-3482 25733754

[B90] Tucha L. Fuermaier A. B. Koerts J. Buggenthin R. Aschenbrenner S. Weisbrod M.. (2017). Sustained attention in adult ADHD: time-on-task effects of various measures of attention. J. Neural Transm. 214, 39–53. 10.1007/s00702-015-1426-0 26206605 PMC5281679

[B91] Tucha L. Tucha O. Walitza S. Sontag T. A. Laufkötter R. Linder M.. (2009). Vigilance and sustained attention in children and adults with ADHD. J. Atten. Disord. 12, 410–421. 10.1177/1087054708315065 18400983

[B92] Uno M. Abe J. Sawai C. Sakaue Y. Nishitani A. Yasuda Y.. (2006). Effect of additional auditory and visual stimuli on continuous performance test (noise-generated CPT) in AD/HD children—usefulness of noise-generated CPT. Brain Dev. 28, 162–169. 10.1016/j.braindev.2005.06.007 16466882

[B93] van Mourik R. Oosterlaan J. Heslenfeld D. J. Konig C. E. Sergeant J. A. (2007). When distraction is not distracting: a behavioral and ERP study on distraction in ADHD. Clin. Neurophysiol. 118, 1855–1865. 10.1016/j.clinph.2007.05.007 17576093

[B94] Vogt C. Williams T. (2011). Early identification of stimulant treatment responders, partial responders and non-responders using objective measures in children and adolescents with hyperkinetic disorder. Child Adolesc. Ment. Health 16, 144–149. 10.1111/j.1475-3588.2010.00593.x 32847239

[B95] Vogt C. Williams T. Susi K. Harrison S. (2018). Differences in measurements of hyperactivity between objective testing using infrared motion analysis (QbTest) and behavioural rating scales when comparing problems in alerting functions and response inhibition during the clinical assessment of ADHD. Psychol. Dis. Res. 1, 3–6. 10.31487/j.PDR.2018.02.002

[B96] Waschbusch D. A. King S. (2006). Should sex-specific norms be used to assess attention-deficit/hyperactivity disorder or oppositional defiant disorder? J. Consult. Clin. Psychol. 74, 179–185. 10.1037/0022-006x.74.1.179 16551155

[B97] Willard V. M. Conklin L. H. Huang L. Zhang H. Kahalley L. S. (2016). Concordance of parent-, teacher- and self-report ratings on the Conners 3 in adolescent survivors of cancer. Psychol. Assess. 28, 1110–1118. 10.1037/pas0000265 27537005 PMC4991558

[B98] Willcutt E. G. (2012). The prevalence of DSM-IV attention-deficit/hyperactivity disorder: a meta-analytic review. Neurotherapeutics 9, 490–499. 10.1007/s13311-012-0135-8 22976615 PMC3441936

[B99] Wolraich M. Brown L. Brown R. T. DuPaul G. Earls M. Feldman H. M. (2011). ADHD: clinical practice guideline for the diagnosis, evaluation, and treatment of attention-deficit/hyperactivity disorder in children and adolescents. Pediatrics 128, 1007–1022. 10.1542/peds.2011-2654 22003063 PMC4500647

[B100] Yang P. Jong Y. Chung L. Chen C. (2004). Gender differences in a clinic-referred sample of Taiwanese attention-deficit/ hyperactivity disorder children. Psychiatry Clin. Neurosci. 58, 619–623. 10.1111/j.1440-1819.2004.01312.x 15601386

