# Supplementary material for: Gender Differences in Objective and Subjective Measures of ADHD Among Clinic-Referred Children
Source: Front Hum Neurosci. 2019 Dec 13;13:441. doi: 10.3389/fnhum.2019.00441 (PMC6923191; doi:10.3389/fnhum.2019.00441)
Supplement: Supplementary file 1 [file Image_1.pdf]

Figure S1: Definition of the time line

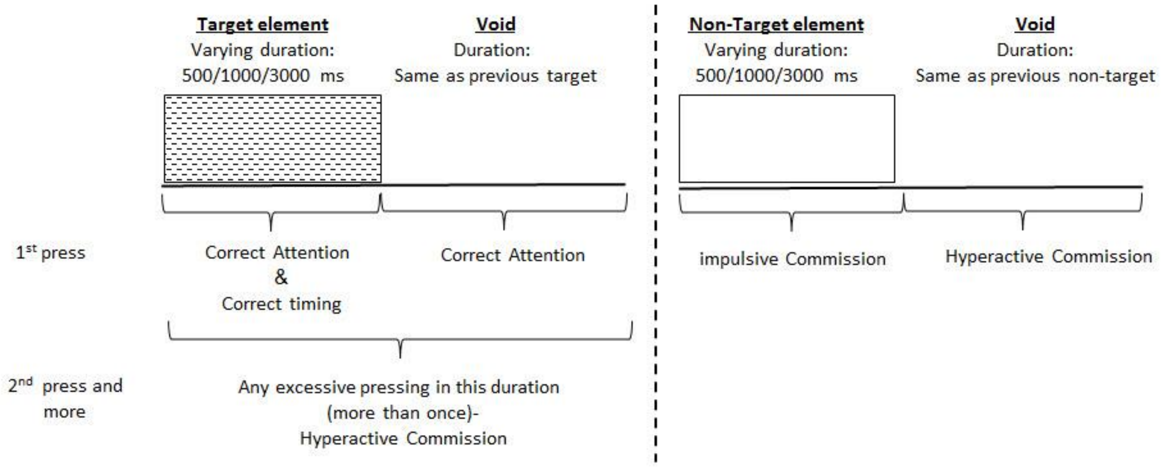

Figure S1 presents the MOXO-CPT task. Target and non-target stimuli were presented for 500, 1000 or 3000 ms in the children's version and 500, 1000 or 4000 ms in the adolescents' version. Each stimulus was followed by a void period of the same duration. The stimulus remained on the screen for the full duration regardless the response. Distracting stimuli were not synchronized with target/ non-target's onset and could be generated during target / non target stimulus or the void period.
