# Supplementary material for: Gender Differences in Objective and Subjective Measures of ADHD Among Clinic-Referred Children
Source: Front Hum Neurosci. 2019 Dec 13;13:441. doi: 10.3389/fnhum.2019.00441 (PMC6923191; doi:10.3389/fnhum.2019.00441)
Supplement: Supplementary file 2 [file Image_2.pdf]

Figure S2: Interaction effect of gender with ADHD symptom type

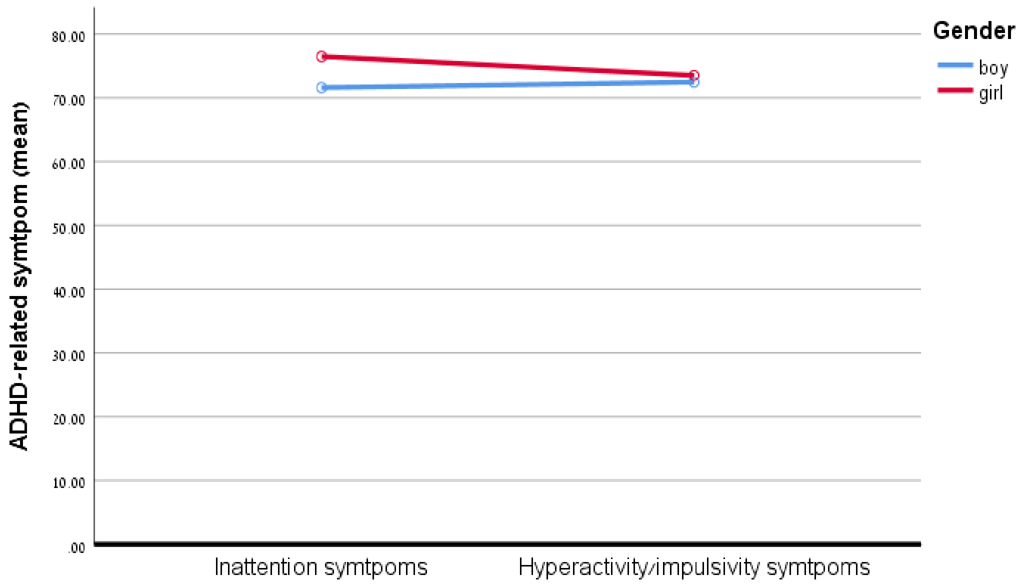

Figure S2 presents the interaction effect between gender and ADHD symptom type according to parents' and teachers' reports (Conners' ADHD rating scales). As can be seen, girls had more inattention problems than boys, but no gender difference was evident in the hyperactivity/ impulsivity symptoms
